# Inhibiting the DNA damage repair of HNSCC cells in combination with normo-fractionated radiotherapy influences clonogenicity, senescence and expression of NK cell activation markers

**DOI:** 10.1038/s41598-025-17858-6

**Published:** 2025-08-29

**Authors:** Tina Jost, Matthias Wachter, Julia Meidenbauer, Rainer Fietkau, Udo S. Gaipl

**Affiliations:** 1 Translational Radiobiology, Department of Radiation Oncology, Universitätsklinikum Erlangen, Friedrich-Alexander-Universität Erlangen-Nürnberg, Universitätsstrasse 27, Erlangen, 91054 Germany; 2 Department of Radiation Oncology, Universitätsklinikum Erlangen, Friedrich-Alexander- Universität Erlangen-Nürnberg, Erlangen, Germany; 3 Comprehensive Cancer Center Erlangen-EMN, Erlangen, Germany; 4 FAU Profile Center Immunomedicine (FAU I-MED), Friedrich-Alexander-Universität (FAU), Erlangen-Nürnberg, Schlossplatz 1, D-91054 Erlangen, Germany; 5 Deutsches Zentrum Immuntherapie, Erlangen, Germany

**Keywords:** Tumour immunology, Senescence, Oral cancer

## Abstract

Treatment of head and neck squamous cell carcinomas (HNSCC) remains challenging with regards to radioresistance, particularly of Human Papilloma Virus (HPV)-negative tumors. Several new approaches are currently under pre-clinical and clinical investigation. Combination of radiotherapy (RT) and kinase inhibitors of the DNA damage repair system (DDRi), targeting Ataxia Telangiectasia Mutated (ATM) or ATM and Rad3-related (ATR), are promising, but the consequences on tumor cell phenotype are still scarce. We used AZD0156, an ATM inhibitor, and VE-822, an ATR inhibitor, in combination with normo-fractionated RT to treat two HPV-positive and two HPV-negative HNSCC cell lines. Generally, an effective reduction of clonogenicity was detected in tumor cells treated with a combination of RT + DDRi. Inhibiting ATM in combination with RT changed the cellular morphology, enhanced β-Gal activity and intensified secretion of senescence-associated cytokines. As senescent cells are naturally targeted by NK cells, we next analyzed the release of the cytokines IL-6 and IL-8 and found them to be differently regulated by the inhibitors. In co-culture with NK cells, an upregulation of activation markers on NK cells was observed, particularly after contact with RT + ATMi-treated HPV-negative HNSCC cells. We conclude that ATM inhibitor-related induction of senescence in HNSCC cells shapes the tumor micro-environment in way that NK cell phenotype is changed.

## Introduction

The gold standard of Head and Neck Squamous Cell Carcinoma (HNSCC) treatment is still a surgical intervention followed by radiochemotherapy (RCT). Based on the tumor origin, predominantly tobacco/alcohol-driven or by infection with Human Papilloma Virus (HPV), the efficiency of this treatment scheme differs remarkably resulting in a worse prognosis for HPV-negative tumors concomitant with reduced overall survival^1^. To improve multimodal treatments several approaches have been investigated pre-clinically and clinically. Besides the implementation of immunotherapy^2,3^, further approaches need to be considered. The use of radiosensitizers, to optimize the radiotherapy (RT) and thereby enhancing the patient’s outcome, is one of them. Radiosensitizers are agents that enhance the sensitivity of cancer cells to radiotherapy, making them more susceptible to radiation-induced DNA damage, e.g. by interfering with cell cycle control or the DNA damage repair machinery. Small molecule kinase inhibitors (smKI) targeting relevant proteins of tumor-associated pathways are currently under scientific focus^4^. Inhibitors of the DNA damage repair (DDR) system should be promising in combination with DNA damage-inducing RT specifically^5^.

According to the hallmarks of cancer, the genome instability and the deregulated cell cycle are, among others, driven by mutations in the DDR system^6^. Pathways for DNA single strand bread (SSB) but also double strand break (DSB) repair, such as homologous recombination (HR) and non-homologous end-joining (NHEJ), are affected by these mutations. Basically, this leads to a mutation-based sensitivity to radiotherapy, which works predominantly via induction of extensive DNA damage by ionizing radiation (IR). Therefore, combining DNA damage-inducing RT with inhibitors of DNA repair pathways represents a promising strategy to overcome radioresistance. Inhibitors targeting key repair proteins, such as Poly(ADP-ribose) polymerase (PARP), which plays a central role in SSB repair, have already been used in clinical practice for several years^7^.

However, there is limited knowledge of the immunogenicity trigged by molecular targeting via kinase inhibitors^8^. Our previous work indicates that the combination of RT and chemotherapy with docetaxel alters the immune phenotype of HNSCC tumor cells, characterized by increased surface expression of CD137-L and release of HMGB1 of specifically HPV-positive tumor cells^9^. Recent work suggests that inhibition of CDK4/CDK6 by p16 in Oral Cavity Squamous Cell Carcinoma (OCSCC) induces senescence in the tumor cells and inhibits DNA repair^10^. But, early senescence is also a determinant of radioresistance in HNSCC^11^. On the other hand, senescent cells can be targeted and eliminated by natural killer cells^12^. How RT in combination with DDRi possibly affects senescence in HNSCC and whether this impacts on natural killer cells, in dependence of the HPV status of the tumor cells, is not known. We therefore hypothesized that inhibition of central proteins of the DDR such as ataxia-telangiectasia mutated protein (ATM) and ataxia telangiectasia and Rad3-related protein (ATR) in HPV-negative and HPV-positive head and neck cancer tumor cells in combination with RT differently affects the immune phenotype of HNSCC cells and consecutively also that of natural killer (NK) cells.

## Results

### Influence of normo-fractionated radiotherapy and smKIs on colony forming

**Fig. 1:**
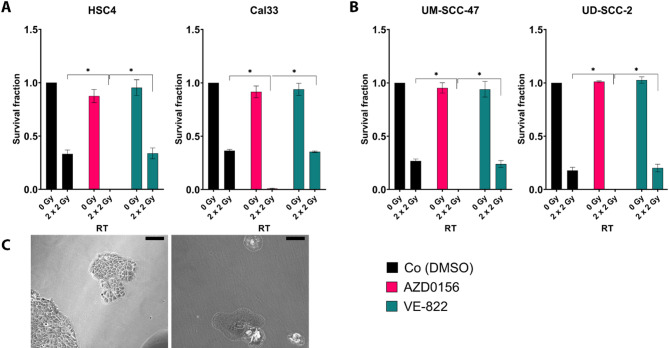
Survival fraction of HNSCC cell lines. **A**) HPV-negative: Cal33, HSC4; **B**) HPV-positive: UM-SCC-47, UD-SCC-2) after treatment with either ATM or ATR inhibitor alone or in combination with 2 × 2 Gy of RT. Normo-fractionated RT decreases clonogenic survival stronger in HPV-positive cell lines. Treatment with smKI alone (ATMi or ATRi) lead to slight reduction of colony forming. But, combination of 2 × 2 Gy and ATMi has strongest reducing effect of clonogenicity. Each value represents mean ± SD (*n* ≥ 4). Significance was calculated by one-tailed Mann-Whitney U Test * *p* ≤ 0.050). **C**) Representative microscopic images of HSC4 cell line 13 days after treatment. Left: Cells treated with 500 nM AZD0156; right: Cells treated with 500 nM AZD0156 + 2 × 2 Gy RT. Scale = 50 μm.

Based on our previously published work, we knew that smKIs of the DDR system can enhance the effect of RT on HNSCC cells in a synergistical manner using 500 nM AZD0156 and 5 nM VE-822^13^. We were now first interested if these effects undergo an alteration when using a clinically more relevant normo-fractionated RT setting. Therefore, enhanced colony forming assays, using conditioned medium, were performed after normo-fractionated irradiation of 2 × 2 Gy in combination with smKIs (ATMi: AZD0156; ATRi: VE-822) using four HNSCC cell lines (Fig. 1).

The survival fraction (sf) of all HNSCC cell lines was hardly reduced by treatment with smKI alone, which is in line with previous findings^13^. The concentrations used here were validated in this preliminary work and showed that clonogenic survival was inhibited to the same extent by these concentrations of AZD0156 and VE-822. Combinatory treatment of 5 nM VE-822 and 2 × 2 Gy RT resulted in similar reduction of sf as normo-fractionated RT alone. Noticeably, the treatment with the ATM inhibitor AZD0156 led to a dramatic decrease of the sf in all cell lines (Fig. 1A). Microscopic observation revealed that the combinatory treatment of ATMi + RT altered the morphology of the cancer cells (Fig. 1B). The extensively increase cytoplasm indicates the establishment of senescence in the treated cancer cells.

To investigate the altered clonogenic behavior of the HNSCC cell lines we used C_12_-FDG staining and the analysis of senescence-associated cytokines in the supernatant to gain greater insides.

### Effect of normo-fractionated radiotherapy and smKIs on senescence of HNSCC

#### ATM inhibitor induced Senescence-associated β-Galactosidase activity (C_12_-FDG staining)

**Fig. 2:**
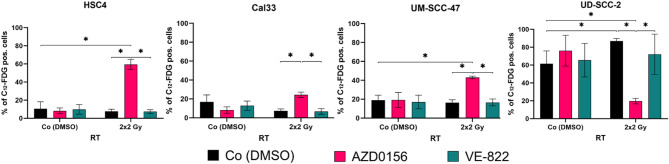
Proportion of C12-FDG positive HNSCC cells after treatment with either ATM or ATR inhibitor alone or in combination with 2 × 2 Gy of RT. Cells were seeded and treated with 500 nM AZD0156 (ATMi), or 5 nM VE-822 (ATRi) and normo-fractionated (2 × 2 Gy) RT. On day 10 post-seeding, cells were stained with C12-FDG and measured via flowcytometry. Each value represents mean ± SD (*n* = 4). Significance was determined by two-tailed Mann-Whitney U Test * *p* ≤ 0.050.

The tumor cells were seeded and treated with smKIs and normo-fractionated RT (2 × 2 Gy) (Fig. 2). On day 10 after seeding, C_12_FDG staining of the HNSCC cells was performed. Monotherapy with either ATMi or ATRi alone did not led to an increase of C_12_-FDG positive cells, an indicator of senescence. HPV-positive cell lines HSC4 and Cal33 as well as HPV-negative cell line UM-SCC-47 showed a comparable outcome with a significant increased amount of C_12_-FDG positive cells after concomitant treatment of RT + ATMi. Highest induction of senescence, was observed in the HPV-negative HSC4 cells. UD-SCC-2 showed a deviating pattern with a high proportion of C_12_-FDG positive cells in general and a drop of senescent cells after RT + ATMi.

#### RT-related increase of a Senescence associated secretory phenotype

**Fig. 3:**
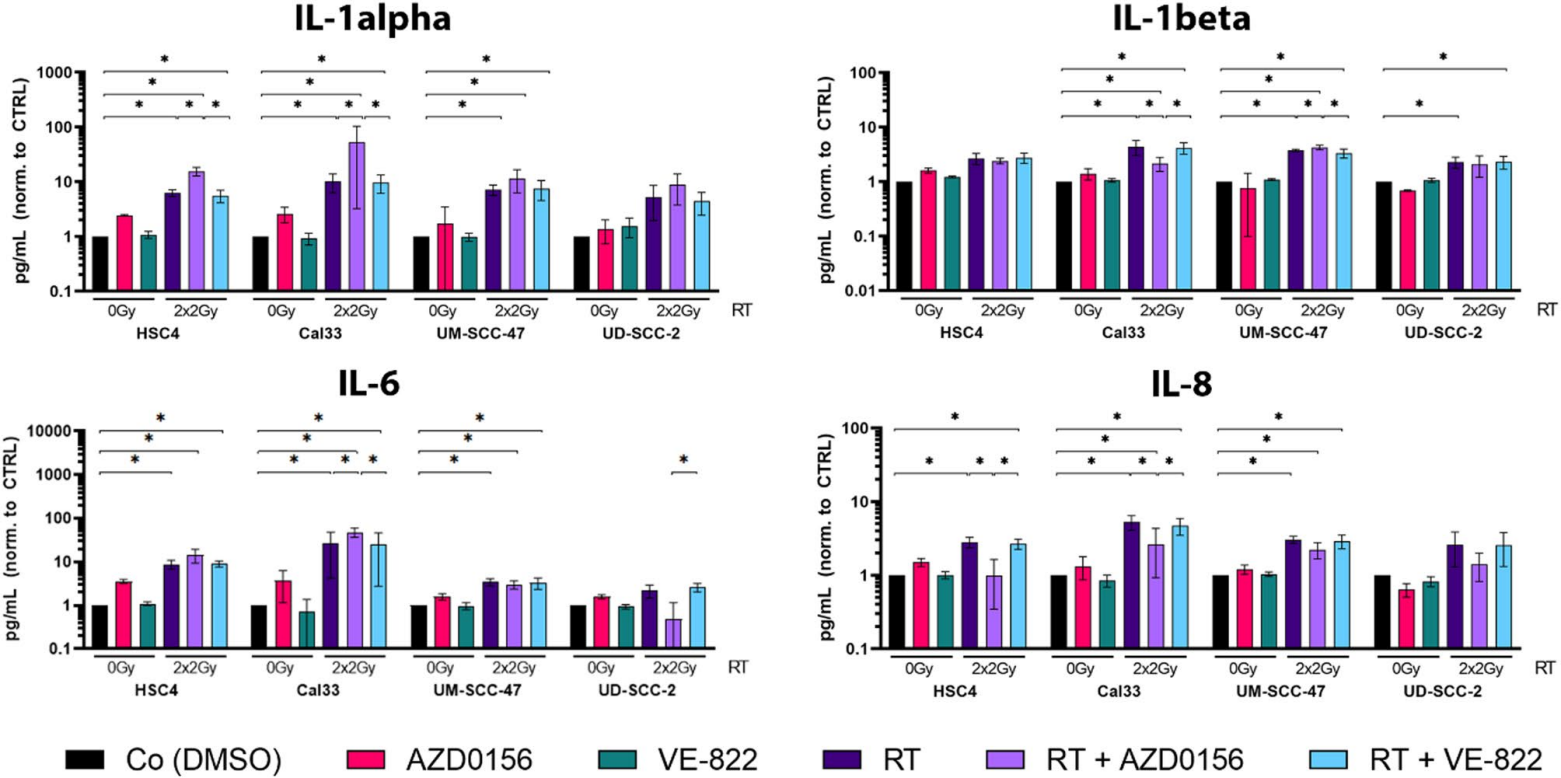
Analysis of secretome of treated HNSCC cell lines regarding the Senescence-associated secretory phenotype (SASP) and pro-inflammatory cytokines. Supernatants were collected from C12-FDG assays 10 days after seeding. Senescence-associated and immunomodulatory cytokines (IL-1α and IL-1β), as well as pro-inflammatory cytokines (IL-6 and IL-8), were quantified using multiplex ELISA kits (MSD). Cytokine levels were normalized to cell confluence of the respective treatment groups relative to the untreated control (DMSO). Subsequently, values were normalized to the DMSO control and expressed as fold change (set to 1). Each value represents mean ± SD (*n* ≥ 3). Significance was determined by one-tailed Mann-Whitney U Test * *p* ≤ 0.050.

The analysis of β-galactosidase activity indicated that ATMi + RT treatment led to establishment of senescence. Furthermore, the cell culture supernatants were analyzed by using Multiplex ELISA, focusing on cytokines known to be associated with the senescence-associated secretory phenotype (SASP). As senescent cells typically secrete cytokines such as IL-1α, IL-1ß, IL-6 and IL-8^11,14,15^, we concentrated our analysis on these factors (Fig. 3).

The secretion of IL-1α, IL-1β, IL-6 and IL-8 was induced by RT in both HPV-positive and HPV-negative cell lines, with the exception of UD-SCC-2. IL-1α levels were consistently either slightly or significantly upregulated in the secretome of ATMi + RT-treated cells. An increased concentration of IL-6 was most prominent in HPV-negative cells treated with ATMi + RT. In general, the secretion of IL-1α and IL-6 was highest in Cal33 cells. Interestingly, IL-8 secretion was downregulated following combined treatment with ATM inhibitor AZD0156 and 2 × 2 Gy RT, in contrast to RT alone or RT + ATR inhibitor (VE-822) treatment.

To get first hints if these alterations might impact on NK cells, we analyzed a tumor cell – NK cell co-culture system.

### Increase of activation markers on NK cells after co-culture with treated HNSCC cells

**Fig. 4:**
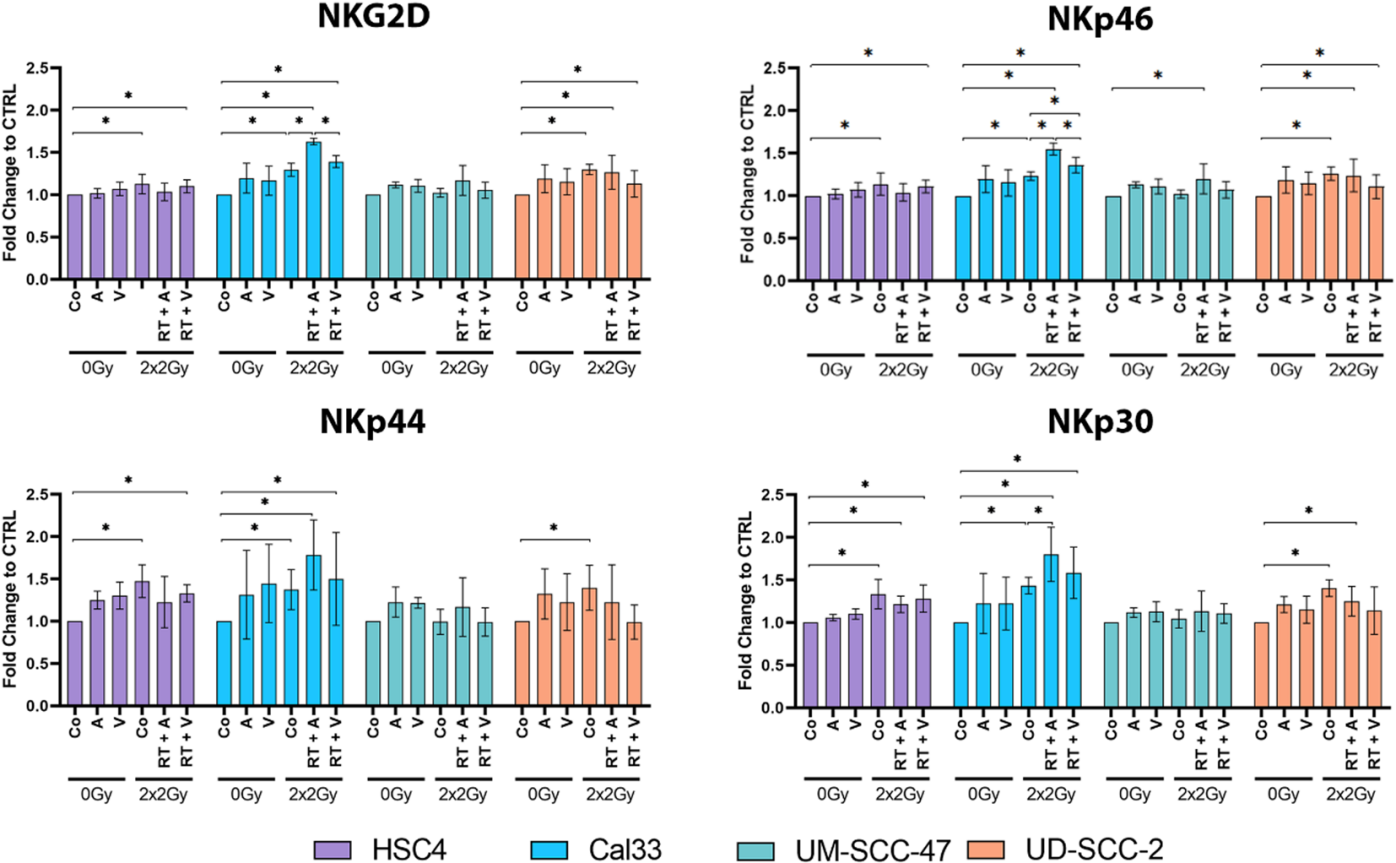
Analysis of activation-associated surface markers on NK cells, co-cultured with pre-treated HNSCC tumor cells. NK cells were isolated from the peripheral blood of healthy donors and stimulated with IL-2 for 72 h. In parallel, HNSCC tumor cells were treated with AZD0156 (A), VE-822 (V) or a combination with 2 × 2 Gy RT. Subsequently, pre-treated tumor cells were co-cultured with IL-2–activated NK cells at an effector-to-target (E: T) ratio of 1:1. After 24 h of co-culture, surface expression of NK cell activation markers—including NKG2D and NKp30/44/46—was analyzed by flow cytometry. Data were normalized to the untreated control (DMSO) and are presented as fold change relative to control. Each value represents mean ± SD (*n* ≥ 3). Significance was determined by one-tailed Mann-Whitney U Test * *p* ≤ 0.050. Exact p-value for all tested comparisons was *p* = 0.050.

As part of the innate immune system, NK cells have several functions in the human body and clearance of abnormal cells such as senescent cells is one prominent of them. Based on our observation of driving cancer cells, treated with normo-fractionated RT + ATMi AZD0156, into a senescent state, we were interested in the response of NK cells to the treated tumor cells. Stimulated NK cells (IL-2) and treated tumor cells were co-cultivated with a Target: Effector (T: E) ratio of 1:1. Established activation markers such as NKG2D, NKp46, NKp44 and NKp30 on the surface of NK cells were measured by multicolor flow cytometry after 24 h of co-incubation (Fig. 4).

Overall, we observed an increased expression of activation markers on NK cells following co-culture with HNSCC cells treated with RT, compared to non-treated tumor cells—except in the case of UM-SCC-47 cells. Notably, differences among the treatment conditions (RT alone, RT + ATMi, and RT + ATRi) were most prominent in the HPV-negative Cal33 cell line. In this context, the combination of RT and ATM inhibition (RT + ATMi) led to a significantly higher expression of NKG2D and NKp46. Similar, although non-significant, trends were observed for NKp44 and NKp30. In contrast, co-culture with HPV-positive tumor cells resulted in a less pronounced effect on NK cell activation marker expression compared to HPV-negative cells. Interestingly, UD-SCC-2 cells exhibited a decreasing trend in activation marker expression across all treatments—RT alone, RT + ATMi, and RT + ATRi—demonstrating a divergent response compared to the other cell lines, consistent with their distinct senescence profile (Fig. 2).

## Discussion

According to their origin, tumors of the head and neck area differ in tumor biology and the underlying mutations. These differences also affect the success of anti-tumor therapies such as radiotherapy, resulting in a reduced radiosensitivity in e.g. HPV-negative tumors. As anti-cancer efficacy of tumor therapies is related to DNA damage of the tumor cells, inhibitors of the DNA damage response are in the focus for improvement of anti-tumor responses in multimodal settings^16^. Alterations in DDR-related genes were shown to be related to the occurrence, development, and treatment of HNSCC^17^. We hypothesized that treatment with DDRi together with fractionated radiotherapy influence the sensitivity of HNSCC tumor cells to RT. Alongside with the major aim to overcome the intrinsic radioresistance, another important issue is to reactivate the anti-tumor mechanisms of the immune system, to not only benefit from the local effect of RT but to induce a systemic anti-tumor response^18^. Based on the synergistic reduction of tumor growth of ATM inhibitor AZD0156 and ATR inhibitor VE-822 with a single dose of 1 × 2 Gy^13^, we first aimed to enhance this effect with a more clinically relevant RT scheme. Since normo-fractionated protocols are the standard of care in radiation oncology, and are discussed to impact on the immune phenotype of HNSCC cells^19^, we focused on testing the combination of ATM and ATR inhibitors with a 2 × 2 Gy RT setting. Further, a 2 × 2 Gy setting ensures a sufficient induction of DNA damage and treatment response while maintaining adequate cell viability. The clonogenic survival was hardly influenced by kinase inhibitors alone, but HPV-positive cell lines showed greater growth inhibition after RT, then HPV-negative cell lines. The sensitivity to RT was significantly enhanced by the combination of smKIs. Predominantly, the ATM inhibitor AZD0156 led to almost complete reduction of proliferation when being combined with 2 × 2 Gy RT (Fig. 1). According to these findings, Kocher et al. also showed increase of yH2AX foci in UD-SCC-2 after 1 × 2 Gy RT under ATM inhibition, indicating the radiosensitization ability of AZD0156^20^. Morphological alterations of the ATMi + RT treated cells indicated an establishment of senescence in the tumor cells. This might be of certain interest, since Schoetz et al. demonstrated that radioresistance mechanisms are also based on RT-induced cellular senescence in HNSCC^11^. Nevertheless, senescence is still controversy discussed in cancer research based on findings underlying a tumor promoting effect versus senescence as key to inhibition of tumor cell growth^11,21^. Further, senescent cells are a target for elimination by NK cells with therapy-mediated cellular stress pathways as trigger to stimulate NK cell effector functions^22^. Generally, therapy-induced senescence has been identified as a key component of tumor biology^23^.

Looking at the effect of ATM inhibitor AZD0156 more closely, revealed an upregulation of senescence-associated β-galactosidase in ATMi + RT treated tumor cells (Fig. 2), emphasizing the discussed establishment of a strong cell cycle arrest with no detectable colony formation (Fig. 1). Of note is the different behavior of UD-SCC-2, which in contrast showed a strong reduction of β-galactosidase after RT + ATMi compared to the control and other treatments being characterized by high senescence levels compared to the other cell lines. The comparatively lower induction of senescence observed in UD-SCC-2 cells following combined radiotherapy and ATM inhibition is attributed to the induction of cell death induced by a pronounced accumulation of cells in the radiosensitive G2 phase. This phase-specific enrichment could potentiate radiation-induced DNA damage, and the concurrent inhibition of ATM may promote a shift in cell fate from senescence toward cell death. Such a mechanism may account for the reduced β-galactosidase activity detected in the combination treatment group, despite the anticipated increase in DNA damage burden^20,24,25^. Interestingly, KRAB-associated protein 1 (KAP-1), an ATM target protein, is stronger phosphorylated after RT in UD-SCC-2 than in UM-SCC-47 and associated with DNA damage response, oncogenic programs and autophagy and could therefore be another factor that explains the significant difference since, both cell lines express wild-type p53^20,26^. The analyses of SASP demonstrated an increase of IL-1alpha, but also of IL-6 (Fig. 3). This upregulation was induced by RT in both, HPV-positive and -negative cells, but intensified by combination of RT + ATMi in HPV-negative cell lines. It is known that senescent cells have a strong influence on cells of the innate immune system, mainly driven through secretion of pro-inflammatory cytokines as a part of the SASP. Two cell types are mainly promoted and recruited by the SASP, macrophages and NK cells^21^. The recruitment of NK cells to the side of senescent tumor cells, leads to expression of NK cell activation marker on their surface and finally to the killing of the senescent cell. Additionally, we recognized a downregulation of IL-8 after combination of RT + ATMi. Chen et al. found that IL-8 is regulated by ATM^27^. We detected a reduced secretion of IL-8 after RT + ATMi treatment compared to RT or RT + ATRi treatment. Further, there is evidence that IL-8 might not only promote HNSCC but also stimulate its activity^28^. In this context, reduction of IL-8 could possibly be beneficial for cancer patients. The observed downregulation of IL-8 following combined radiotherapy and ATM inhibition is particularly interesting given IL-8’s roles in promoting tumor growth, angiogenesis, and immune evasion, including suppression of NK cell function. An important consideration regarding IL-8 regulation is the interplay between DNA damage, ATM signaling, and inflammatory pathways. ATM has been shown to activate NF-κB signaling in response to DNA double-strand breaks^29^, and NF-κB serves as a central transcriptional regulator of inflammatory mediators, including IL-8^30^. Moreover, DNA damage is known to induce the production of reactive oxygen species (ROS)^31^, and this effect can be amplified in the presence of ATM inhibitors. Since ROS can stimulate IL-8 expression through NF-κB activation^32^, the observed downregulation of IL-8 after ATM inhibition in combination with radiotherapy may reflect disrupted ROS-NF-κB signaling. These mechanistic links suggest that ATM inhibition modulates the inflammatory tumor microenvironment and could potentially influence immune effector function. While our study focused on phenotypic markers of NK cell activation, we did not directly assess whether reduced IL-8 levels contribute to enhanced NK cell cytotoxicity. As such, the functional link between IL-8 modulation and NK cell-mediated clearance of tumor cells has to be investigated in the future. Experiments employing IL-8 neutralization or supplementation could help elucidating whether IL-8 is a critical mediator of NK cell activity in this context.

A limitation of this study is the absence of direct functional evidence demonstrating NK cell-mediated cytotoxicity. But, the observed upregulation of several key activation markers—including NKG2D and NKp46—provides valuable indirect evidence suggestive of enhanced NK cell responsiveness. It is important to note that while these receptors alone may not be sufficient to trigger cytotoxicity, their co-expression and synergistic activation are known to play a critical role in effective NK cell-mediated tumor cell killing. Future studies incorporating cytotoxicity assays, such as LDH release or Annexin V/PI staining, are necessary to validate whether NK cells effectively eliminate senescent tumor cells under these conditions. In our study senescence was primarily assessed by changes in cell morphology and β-galactosidase activity (C12-FDG). The inclusion of additional molecular markers such as p16^INK4A^, p21^Cip1^, or senescence-associated heterochromatin foci (SAHF) should be envisaged in future analyses. While the NK cell–tumor cell co-culture model employed in this study enables a controlled investigation of direct immune-tumor interactions, it does not fully recapitulate the complexity of the in vivo tumor microenvironment. Factors such as the presence of additional immune cell subsets, extracellular matrix components, and the influence of stromal and vascular compartments are not represented in this in vitro system. Therefore, future studies using in vivo models or 3D tumor spheroids will be essential to confirm and expand on the immunomodulatory effects observed in this setting.

In our chosen preclinical model system, we co-cultured HNSCC tumor cells with, to focus on the ability of the treated tumor cells to alter the activation of NK cells in the near surrounding. Wu and colleagues previously demonstrated that IL-6 and IL-8 secreted by tumor cells play a crucial role in the weakening of NK cell in the context of HNSCC^33^. We observed an upregulation of NKG2D, NKp46, NKp44 and NKp30 in 3 out of 4 cell lines (Fig. 4). Cal 33 tumor cells treated with the combination of ATMi + RT significantly increased the expression of activation receptors on NK cells. This suggest that ATMi + RT is capable of indirectly stimulating NK cell responses against HNSCC cells.

Our in vitro findings demonstrate that ATM inhibition, particularly in combination with radiotherapy (RT), significantly reduces tumor cell survival and enhances cellular senescence, suggesting a potential strategy to increase the efficacy of RT in HNSCC. These results have translational relevance, as ATM status may serve as a predictive biomarker for response to DNA damage–based therapies. Patients with tumors exhibiting high ATM activity may particularly benefit from pharmacologic ATM inhibition to sensitize cancer cells to RT-induced DNA damage. Moreover, the observed induction of senescence raises the possibility of using senescence-associated markers (e.g., p21, β-galactosidase activity, or SASP cytokines) as indicators of therapeutic response. However, given the complex role of therapy-induced senescence, including its potential to promote tumor progression via SASP, further preclinical and clinical studies are needed to assess whether combining ATM inhibitors with senolytic agents could enhance treatment outcomes while minimizing pro-tumorigenic side effects.

## Conclusion

Our data suggest that combination of ATMi plus fractionated RT very efficiently reduces clonogenicity in HNSCC and induces senescence that is capable of activation NK cells. This is not predominantly determined by the HPV status of the tumor cells, but rather an individual feature, in part depended on SASP and secreted cytokines. The combination of DDR inhibitors and radiotherapy is still one promising option in multimodal treatment of head and neck cancer cells, to overcome challenges such as radioresistance.

## Material & method

### Culture of head and neck squamous cell carcinoma cells

The head and neck tumor cell lines HSC4, Cal33 (HPV-negative) and UM-SCC-47, UD-SCC‑2 (HPV-positive) were kindly provided by Dr. Thorsten Rieckmann (Hamburg, Germany), and cultured in DMEM medium (Pan Biotech) containing 10% fetal bovine serum (FBS, Sigma-Aldrich) and 1% penicillin/streptomycin (PenStrep, GIBCO Life Technologies, Thermo Fisher Scientific, Waltham, MA, USA). Natural killer (NK) cells from healthy human donors (isolation procedure described below) were cultured in RPMI1640 medium (Sigma-Aldrich, St. Louis, MO, USA) containing 10% FBS, 1% Pen/Strep and 100 U/mL IL-2 (Peprotech, Hamburg, Germany) for stimulation. Cell culture was incubated under standard conditions at + 37 °C, 5% CO_2_, and 95% humidity (Incubator RBP 6220, Hereus Instruments, Hanau, Germany).

### Kinase inhibitor treatment and irradiation setting

Cells were treated with the ATM inhibitor AZD0156 (Selleckchem, Houston, TX, USA) or the ATR inhibitor VE-822 (Selleckchem, Houston, TX, USA), diluted in dimethyl sulfoxide (DMSO, Roth, Karlsruhe, Germany) and stored at −80 °C. Subsequently, cells were treated with either 500 nM AZD0156 or 5 nM VE-822 for 24 h after seeding. As a vehicle control (Co, CTRL) cells were treated with DMSO alone. Cells were irradiated according to a normo-fractioned scheme with a dose of 2 × 2 Gy (Gy) 3 h after treatment with smKI and with second dose after 24 h using the ISOVOLT Titan X-ray generator (GE, Ahrensburg, Germany). Cells and supernatant were collected for analysis 48 h post-RT.

### Colony forming assays with conditioned medium

Colony forming assays were performed as previously described^13^ and further optimized by using conditioned medium according to Brix et al.^34^. Briefly, a single cell suspension was prepared by trypzination. Cells were counted and seeded into 6-well-plates in a defined number of cells (250–500 cells/well w/o RT; 1000–2000 cells/well with RT) depending on the treatment conditions in order to ensure optimal colony growth and accurate quantification of clonogenic survival. Wells were filled with 1:1 fresh D10 (DMEM + 10% FBS + 1% Pen/Strep) and conditioned D10 medium. Conditioned D10 was prepared by seeding a suitable number of cells into a T175 cell culture flask, containing 50 mL D10, to reach at least 70% confluence after 72 h of incubation. The supernatant was collected and centrifuged (300xg, 10 Min) and 45 mL were carefully collected from the tube. The medium was frozen at – 80 °C and immediately thawed prior to seeding of a new experiment. Cells were treated with AZD0156 or VE-822 24 h after seeding (day 1) and irradiated 3 h after treatment. Second radiation dose was given 24 h (day 2) after the first dose and medium was exchanged on day 3 (24 h post-RT). Plates were incubated for 12 to 14 days until colonies, including 50 cells in minimum, were microscopically detectable. Finally, the medium was collected and stored at – 80 °C and cells were washed, stained with eosin-methylene blue solution for 45 Min and counted. Survival fraction (sf) was calculated accordingly to the plating efficiency (PE).

### Senescence analysis

The HNSCC cells were seeded and treated according to our colony forming protocol. The cells were harvested 8 days post-RT, centrifuged, and resuspended in 400 µL of D10 followed by staining with 4 µL of 10 µM bafilomycin-A1 solution and then incubated for 1 h at + 37 °C. Afterwards, 0.5 µL of 20 nM 5-Dodecanoylaminofluorescin-di-beta-D-galactopyranoside (C_12_-FDG) was added and then incubated again for 1 h hour. After centrifugation (300 x g, 5 Min), cells were resuspended in 100 µL of Ringer solution containing Annexin V-FITC (AxV, 0.5 µg/mL) and 1 µL of Proprium Iodid (PI, 1.0 µg/mL) solution. Cells were incubated for 30 Min (+ 4 °C) in the dark. Finally, the cells were again centrifuged and resuspended in 100 µL Ringer solution and analyzed by flow cytometry (Cytoflex S, Beckman Coulter, Beas, USA).

### Multiplex Enzyme-Linked immunosorbent assay (ELISA)

The concentration of senescence-related cytokines in 1 mL of supernatant from the C_12_-FDG senescence analyses experiments was measured using a Multiplex ELISA Kit from Meso Scale Discovery (MSD) targeting several cytokines, that are associated with the Senescence Associated Secretory Phenotype (SASP) such as IL-1α and IL-1ß as well as strong immune-modulators such as IL-6 and IL-8. Assay was performed according to the protocol provided by the manufacturer. Samples were not diluted before measuring and collected in four independent experiments (*n* = 4). The mean values ± standard deviation (SD) were used for statistical analysis. Fluorescence intensity was measured for the standard solution, provided by MSD, and all samples. Further, confluence of all sample wells was documented and use for normalization of fluorescence values according to the confluence of each sample well compared to the control (untreated) samples. Finally, the normalized fluorescence values were additionally normalized to the control (set to “1”).

### Activation of NK cells and NK cell - tumor cell co-culture

Peripheral blood mononuclear cells (PBMCs) were isolated from healthy donor blood samples according to our previous work^35^. NK cells were isolated from PBMCs using the NK cell isolation kit (human) from Miltenyi (Ref.Nr. 130-092-657; Miltenyi Biotec B.V. & Co. KG, Bergisch-Gladbach, Germany) based on a negative selection. Isolated NK cells were stimulated using 100 U/mL IL-2 in RPMI 1640 and incubated for at least 72 h.

In parallel, tumor cells were seeded in 6-well-plates and treated according to our colony forming protocol. Inhibitor treatment and first RT dose on day 1, second RT dose on day 2 and medium exchange on day 3. On day 3 the stimulated NK cells were added to the tumor cell culture with a target: effector (T: E) cell ratio of 1:1 and co-culture was incubated for 24 h^36^. Afterwards, detached NK cells and attached tumor cells were harvested and stained for surface marker correlated with activation status of NK cells (Table 1).

**Table 1:** Antibodies used for flow cytometric analysis of NK cell activation.

Antibody	Target	Ref. number	Company
Human Anti-CD314 APC-conjugated	NKG2D	FAB139A	R&D Systems, Minneapolis, MN, USA
Human Anti-CD335 PC5-conjugated	NKp46	A66902	Beckman Coulter, Indianapolis, IN, USA
Human Anti-CD336 BV421-conjugated	NKp44	744,299	BD Bioscience, Heidelberg, Germany
Human Anti-CD337 Alexa Fluor^®^ 594-conjugated	NKp30	FAB1849T	R&D Systems, Minneapolis, MN, USA

### Statistics

For statistical analysis GraphPad prism 9 software (San Diego, CA, USA) was used. One-tailed and two-tailed Mann–Whitney U test was performed and a *P*-value ≤ 0.050 was determined as significant. Corresponding graphs were generated also by GraphPad Prism 9 software.

### Consent

Blood cells were isolated from leukoreduction system chambers (LRSC) of healthy, anonymous donors having undergone a strict health check by the Transfusion Medicine and Hemostaseology Department of the Uniklinikum Erlangen (Germany). The permission to use this LRSC was given by the institutional review board at Friedrich-Alexander-Universität Erlangen-Nürnberg approved the study (No. 48_19 B, approved on 12th September 2019). The study was performed in accordance with the Declaration of Helsinki. All patients gave written informed consent that comprised a data privacy clause for data collection and analysis for research purposes.

## Data Availability

The datasets generated or analyzed during this study are available from the corresponding author upon reasonable request.

## Abbreviations

ATM: Ataxia-Telangiectasia mutated
ATMi: ATM Inhibitor
ATR: Ataxia telangiectasia and Rad3-related
ATRi: ATR Inhibitor
C12-FDG: 5-Dodecanoylaminofluorescein Di-β-D-Galactopyranoside
DDR: DNA damage response
DDRi: DNA Damage response inhibitor
DSB: Double-strand break
ELISA: Enzyme-linked immunosorbent assay
HNSCC: Head and neck squamous cell carcinoma
IL-2/6/8: Interleukin-2 / Interleukin-6 / Interleukin-8
KAP-1: KRAB-associated protein 1 (also known as TRIM28)
NK: Natural Killer (cells)
NKp30/44/46: Natural cytotoxicity receptors (NKp30, NKp44, NKp46)
NKG2D: Natural killer group 2 member D
OCSCC: Oral cavity squamous cell carcinoma
RCT: Radiochemotherapy
RT: Radiotherapy
SAHF: Senescence-associated heterochromatic foci
SASP: Senescence-associated secretory phenotype
smKI: Small molecule kinase inhibitor
SSB: Single-strand break
